# Overcoming the Digital Divide in the Post–COVID-19 “Reset”: Enhancing Group Virtual Visits with Community Health Workers

**DOI:** 10.2196/27682

**Published:** 2021-07-08

**Authors:** Megha K Shah, Ashley Christina Gibbs, Mohammed K Ali, K M Venkat Narayan, Nadia Islam

**Affiliations:** 1 Department of Family and Preventive Medicine School of Medicine Emory University Dunwoody, GA United States; 2 Hubert Department of Global Health Emory Rollins School of Public Health Emory University Atlanta, GA United States; 3 Division of Population Health Grossman School of Medicine New York University New York City, NY United States

**Keywords:** community health workers, COVID-19, diabetes mellitus, eHealth, elderly, health equity, telemedicine, virtual, vulnerable populations

## Abstract

The COVID-19 pandemic created numerous barriers to the implementation of participant-facing research. For most, the pandemic required rapid transitioning to all virtual platforms. During this pandemic, the most vulnerable populations are at highest risk of falling through the cracks of engagement in clinical care and research. Nonetheless, we argue that we should reframe the discussion to consider how this transition may create opportunities to engage extensively to reach populations. Here, we present our experience in Atlanta (Georgia, United States) in transitioning a group visit model for South Asian immigrants to a virtual platform and the pivotal role community members in the form of community health workers can play in building capacity among participants. We provide details on how this model helped address common barriers to group visit models in clinical practice and how our community health worker team innovatively addressed the digital challenges of working with an elderly population with limited English proficiency.

## Introduction

The rapid adoption and escalation of telemedicine during the current COVID-19 pandemic has led to concerns about widening gaps in health equity [1]. Among populations with limited digital access or literacy, notable gaps in access to care have been found; for example, less than one-third of Medicare beneficiaries aged over 65 years have reported digital access at home, and those aged over 75 years and with less than high school–level education are less likely to use technology for health care needs [2,3]. Potentially vulnerable groups include underserved racial and ethnic groups, older adults, and adults with limited English proficiency (LEP), for whom barriers to care access and health information technology have been well-documented, exacerbated by the pandemic, and are at risk of exacerbation [3-8].

In addition, the pandemic and social distancing guidelines have profoundly limited the ability of vulnerable populations to access reliable health care [9,10]. Particularly for chronic disease prevention and management, this can lead to delays in care, which may result in poor health outcomes. Over 40% of respondents in a recent survey of over 5000 adults in the United States reported delaying or avoiding care owing to concerns related to COVID-19, which has been linked to excess deaths reported in 2020, compared to prior years [9]. Older adults, people of color, and low-income individuals—the same populations with the lowest telemedicine access and literacy—have been disproportionately afflicted by COVID-19 and are at the highest risk of cardiometabolic disease [11,12]. The confluence of health risk and the digital divide in these communities creates an environment in which these groups may be most vulnerable to gaps in care and social isolation, which are factors known to exacerbate chronic health conditions.

Despite these challenges, virtual group visits led by community health workers (CHWs)—trained public health professionals with shared lived experience of the communities they serve—may be a pivotal strategy to help address the digital divide in telehealth and foster social connectedness. Social connections can not only influence the risk of chronic illness but also improve chronic disease management [13,14]. Social support is considered a major component of chronic disease self-management [15]. Previous studies suggest that adults with limited social interactions are less likely to engage in health behaviors such as physical activity, smoking cessation, and healthy eating habits [16]. To address this and stimulate socially cohesive and supportive care, group visits have emerged as a type of visit format in which a portion of the visit is a group education class, which can be led by a health coach, nutritionist, or medical assistant, and a portion of time is spent in a brief one-on-one visit with a clinician. Nonetheless, the implementation of group visits has some challenges [17,18]. These challenges include visit-level challenges such as the logistics of clinic workflow and meeting space, patient-level factors such as scheduling or transportation conflicts, and group-leader level challenges such as comfort level in a group role and engaging and retaining participants.

Group visits can be enhanced by meeting patients where they live, work, and worship. CHWs are trained and trusted members of their community, who can serve as a bridge between clinical and community settings. CHWs can work in a variety of capacities to support health education, treatment adherence, health system navigation, and linkage to social services for some of the most vulnerable populations with chronic health conditions [19]. Integrating CHWs into and having them lead group visits may be a particularly effective approach to engaging patients in socially cohesive activities because of CHWs’ familiarity with the communities that they work with. Often, they have shared experiences that enhance rapport-building with patients. A systematic review reported that chronic disease management interventions that included CHWs were associated with increases in tobacco cessation, improved blood pressure control, and low blood sugar levels, with no risk of adverse events [20].

In the current context of the COVID-19 pandemic, virtual group visits may have additional challenges but also expose unanticipated advantages to address the digital divide. Here, we describe our experience transitioning from in-person to a virtual CHW-guided group visit intervention for diabetes and hypertension management among older South Asian adults with LEP.

## DREAM Atlanta

Funded by the National Institutes of Health, the DREAM Atlanta study was designed to test the effectiveness of CHW-delivered in-person health education on diabetes and hypertension, which was tailored for South Asian adults living in Atlanta. Over the course of 6 months, those in the intervention group were intended to receive 5 group education sessions and 2 one-on-one home visits. Initiated in the fall of 2019, the pandemic forced the program’s transition to virtual group visits immediately at the start of recruitment. The transition to a virtual program necessitated not only training of CHWs and other study staff in utilizing technology to deliver remote sessions, but also to educate study participants to utilize digital devices and access virtual platforms. Among the 190 adults of South Asian descent enrolled in the program, all reported English as a second language. At baseline, participants were on average, 56 (range 30-80) years old, approximately 40% of whom were over the age of 60 years; 56% were female; 44% were male; and 96% reported having access to a smartphone or tablet device.

The CHWs worked one-on-one with participants to address barriers to engaging with remote technologies such as creating email accounts, downloading apps on smart devices, and teaching participants how to use features of videoconferencing such as the video and mute features. CHWs utilized several strategies to help participants connect.

## Rapid Transition Assessment Methods and Findings

To gather the perspectives of the study team on the transition to virtual group visits, a 90-minute, tape-recorded video group discussion was conducted with 3 CHWS and the study coordinator. Discussion topics included a discussion of feasibility to conduct virtual recruitment and study sessions, barriers to implementing the study virtually, and specific adaption processes utilized. These data were supplemented with reviews of meeting minutes from weekly team meetings held from March to December 2020. The first and senior authors reviewed common themes emerging from the group discussion and reviews of meeting minutes to determine key facilitators and barriers to the implementation of the remote intervention. All study activities conducted were reviewed by the institutional review board of the New York University School of Medicine, which served as the single institutional review board for this study.

Table 1 lists the digital challenges and solutions fostered by the CHW team. For example, CHWs would start with technologies that participants were familiar with to help them learn to log on to the videoconferencing apps. Most of the DREAM study participants accessed the internet on their smartphones, and similar to studies in South Asia, participants report familiarity with platforms such as Facebook and WhatsApp [21]. Thus, for example, if participants used WhatsApp, the CHW would ask them to join a video call on this platform to help them understand the process of connecting to study-approved remote platforms such as Zoom. This teach back method by the CHWs to their participants greatly enhanced their confidence to schedule and lead group virtual visit sessions.

In the 5 months of this transition, we have observed CHWs’ unique strengths to help close the digital divide in this highly vulnerable population. While previous studies have demonstrated the effectiveness of mobile technologies for chronic health conditions, including diabetes self-management, few studies have examined or reported on the process of getting participants connected to telehealth platforms [22,23]. However, technical challenges and education of patients about telehealth services are well-documented challenges for health systems’ implementation of telehealth services [24]. Similar to our experience, a recent systematic review identified participant training in videoconferencing by an information technology specialist or a group facilitator as a method to overcome participant challenges to connect for psychotherapy interventions [25]. Thus, our findings add to and may have broader implications to address the known challenges of user-related technical difficulties associated with virtual health services.

**Table 1:** Digital challenges and strategies used by community health workers to enhance attendance in virtual group visits among South Asian adults with limited English proficiency.

Digital challenge	Strategy or modification	Example or representative quote
Community health workers had limited experience with videoconferencing software	In-person sessions with the project coordinator and trial and error with the platform to learn to use features	“The project coordinator met with us [the CHWs] in the office and we created the initial set up and user experience for zoom.” “There was steady progress with features such as using the chat box, chatting, and muting participants as the CHWs became more familiar with interface [sic].”
Participants did not know how to download apps on their smartphone	Call the participant or involve a family member and walk them through the steps to download the app Provide in-language and empowerment to help participants address frustrations with new technology	“[We] encourage the participant that a challenge they experience is common (such as problems with the audio), and this encourages the participant to continue to work through the technical situation.”
Challenges with scanning consent forms or other survey documents	Provide real-time virtual or telephone assistance to troubleshoot issues with documents rather than waiting for weekly meetings or follow-up meetings or calls	“At the time that the CHW/participant needs to send a form, they call the coordinator/CHW to learn the skill. The person receiving the form then sometimes makes quick edits to the form to make it legible or printable.”
Participants did not have email addresses to receive study documents	Involve family members who can provide email addresses or support participants Choose a communication strategy that the participant might be familiar with, such as a text messaging platform	“It is often easier to send a link through text message or WhatsApp than email. In general, email proficiency of the participants has not change over time. It is not seen as an easy form of communication in the community.”
Participants did not know how to log in to Zoom	Involve family members in the meetings Schedule meetings on the basis of the availability of other family members	“We ask family members when they will be available and try to schedule sessions at those times.”
Internet connectivity challenges	Be flexible with scheduling and offer assistance at multiple times and days of the week, including weekends and evenings to attend sessions Change to audio only or telephone sessions if participants cannot connect	“Multiple family members may be using the same smartphone for internet; thus, we offer our sessions on the weekend and evenings and often have to reschedule to make sure participants can attend [sic].”

Moreover, they have addressed several known challenges of group visits, which are both participant- and visit-related. Firstly, the barrier of adequate transportation, particularly acute in large, diffuse urban areas including Atlanta—which has limited public transportation options—can prevent participants from attending group visits. Previous studies on barriers to participation in group medical visits include the lack of transportation or scheduling conflicts [26,27]; nonetheless, by offering this virtual option, participants report that this has facilitated their ability to engage in the intervention. One female CHW commented, “For our participants, Zoom is better, because they don’t need to get ready or drive anywhere. We are able to spend more time with them.”

Logistical issues of meeting spaces and workflow can lead to inefficiency and be time-intensive for group visit implementation in clinical practice [27,28]. The transition to virtual visits has provided flexibility to the team for scheduling and location, which has likely enhanced participation and engagement and improved the efficiency of the visits. The CHWs report having more flexibility to conduct multiple sessions, if needed, to facilitate participation, as these logistical obstacles are easier to overcome in virtual sessions.

Lastly, all participants share a common background and health conditions. Shared personal health characteristics can greatly enhance participation and patient activation, leading to improved benefits and health outcomes from group visits [26,28]. While still in progress, the retention rate is >90% toward the fifth month of this 6-month program. At the height of the pandemic, at a time when older adults of underserved racial and ethnic communities with LEP were at the greatest risk of social isolation, the virtual group visits have provided much welcomed social interaction for these at-risk individuals. One female CHW commented, “They [participants] are bored at home; now they are eagerly awaiting our sessions!”

While this pandemic has certainly strained most aspects of well-being for a majority of individuals, there are opportunities to shape our “new normal.” Namely, for communities of color, older adults, and those with LEP, where the risk of the digital divide and social isolation may widen, investing in resources, including CHW-led group virtual visits, may help address gaps in care. The group visit model offers the opportunity to enhance social interaction and patient activation, and preliminary results from our study indicate that the virtual group visit is feasible and acceptable for older adults with LEP. Further, we believe that the involvement of CHWs is critical to this process, to help bridge the digital void that is at risk of widening further during this ongoing pandemic and to enhance the digital literacy of our most vulnerable populations. Beyond the current pandemic, we believe that this model is feasible and can continue to reach our most vulnerable populations in the future.

## Abbreviations

CHW: community health worker
LEP: limited English proficiency
